# Factors associated with pressure ulcer and dehydration in long-term care settings in Ontario, Canada

**DOI:** 10.1371/journal.pone.0297588

**Published:** 2024-01-31

**Authors:** Mary Crea-Arsenio, Andrea Baumann, Valentina Antonipillai, Noori Akhtar-Danesh

**Affiliations:** 1 Global Health, McMaster University, Hamilton, Ontario, Canada; 2 School of Nursing, McMaster University, Hamilton, Ontario, Canada; 3 Department of Health Research Methods, Evidence and Impact, McMaster University, Hamilton, Ontario, Canada; Flinders University, AUSTRALIA

## Abstract

Pressure ulcers and dehydration are common conditions among residents of long-term care facilities that result in negative health effects. They have been associated with signs of neglect and increased 30-day mortality among LTC residents. However, they are both preventable and with proper care can be effectively managed and treated. We conducted a retrospective cohort study to examine factors associated with pressure ulcers and dehydration among long-term care residents in the province of Ontario, Canada. Results indicated that close to one-fifth of residents were dehydrated (17.3%) or had a pressure ulcer (18.9%) during the study period. Advanced age was significantly associated with the presence of pressure ulcers and dehydration for both men and women. However, men were more likely to present with a pressure ulcer while women were more likely to exhibit symptoms of dehydration. Study findings also demonstrate the presence of both conditions being higher in municipal and not-for-profit homes compared to for-profit homes. The significant differences observed in relation to home ownership which require further investigation to identify the most relevant factors in explaining these differences. Overall, pressure ulcers and dehydration are preventable conditions that warrant attention from policymakers to ensure quality of care and resident safety are prioritized.

## Introduction

Long-term care (LTC) homes play a critical role in providing essential healthcare services to residents. In Canada, LTC homes are regulated by the government through legislation that sets standards for care. Despite these regulations, numerous reports of poor quality care have been documented over time [1]. Indicators are used to assess the level of care provided to residents. Data are collected through the Resident Assessment Instrument-Minimum Data Set (RAI-MDS) and used to monitor residents in LTC [2]. Two important measures of quality are the development of pressure ulcers and dehydration [3]. These common conditions are preventable and can result in significant negative health effects for residents [4–7]. Moreover, they have been associated with signs of neglect and increased 30-day mortality among LTC residents [8–10]. It is vital to understand the factors associated with pressure ulcers and dehydration to identify the burden on care provision in LTC homes across Canada. Using resident-level data, this study examined the key predictors of pressure ulcers and dehydration among LTC residents in Ontario, Canada’s most populous province.

Research has focused on individual and organizational factors associated with the development of pressure ulcers and dehydration, both of which are prevalent among LTC residents [6, 11]. Edsberg et al. (p.586) defined pressure ulcers as “localized injury to the skin and/or underlying tissue, usually over a bony prominence, as a result of pressure, or pressure in combination with shear" [12]. Advanced age is a significant risk factor for pressure ulcers, with residents over 80 years of age being at a higher risk [13]. Residents with medical comorbidities such as diabetes and cardiovascular disease and those with low BMI and poor nutritional status, including malnutrition, are also at a higher risk [5, 14, 15]. Incontinence and immobility, including the inability to move and turn independently, are additional risk factors [16, 17].

Similar to pressure ulcers, causes of dehydration are multifactorial. Dehydration occurs when the body loses more fluids than it takes in thus leading to a disruption of normal functions [18]. Older adults are at a higher risk of dehydration due to age-related changes in the body such as decreased sense of thirst and decreased ability to conserve water [4, 18, 19]. The use of certain medications such as diuretics can also increase the risk of dehydration [19]. Moreover, residents with mobility limitations may have difficulty accessing water sources [20]. Chronic illnesses such as diabetes, dementia, and heart failure can increase the risk of dehydration by altering the body’s fluid balance [21, 22]. For example, people with diabetes may experience increased urination due to high blood glucose levels. Those with dementia may have difficulty communicating their thirst or may forget to drink fluids [6].

The literature identifies organizational factors such as inadequate staffing and home ownership (i.e., for-profit versus not-for-profit) as factors associated with pressure ulcers and dehydration. Adequate staffing levels, measured as the ratio of staff to residents, are essential to ensure residents receive timely and appropriate care [23]. In Canada, the government sets staffing standards depending on the level of care required. The minimum staffing standard for LTC homes in Ontario is 4.0 hours of direct care per resident per day [24]. Studies have documented that higher nursing staff levels are associated with lower rates of pressure ulcers in LTC homes [25, 26]. Similarly, higher levels of staff can reduce the risk of dehydration, as staff can monitor and encourage residents to consume fluids more regularly [4].

It is well documented that staffing levels vary according to home ownership. Not-for-profit homes have higher staffing levels and better health outcomes compared to for-profit homes [27, 28]. Inadequate staffing can lead to neglect of LTC residents, while inadequate supervision can lead to increased risk of pressure ulcers and dehydration due to delayed recognition and reporting of symptoms. In a recent study by Akhtar-Danesh et al. [8], pressure ulcers and dehydration were examined to assess the relationship between clinical indicators of neglect and mortality among LTC residents. Results indicated that residents living in for-profit homes had 18% increased risk of dying from neglect compared to those living in government-run homes.

A further understanding of the predictors of pressure ulcers and dehydration is needed to identify residents who are at a higher risk and to develop strategies to reduce the incidence and consequences. The latter include increased burden of care for providers as well as health complications and reduced long-term survival for residents. We conducted a retrospective study to examine the factors associated with pressure ulcers and dehydration among residents of LTC homes in Ontario using individual- and organizational-level data. This approach differs from previous studies that have used individual-level data only.

## Materials and methods

### Data sources

This study included data from the residents of LTC homes in Ontario for the period of January 2019 to February 2020. We used population-based health administrative datasets available through the Institute for Clinical Evaluative Sciences (ICES). We also used the RAI-MDS 2.0 dataset which includes information on clinical, functional, and psychosocial characteristics of LTC residents. The datasets were accessed in July 2022 for research purposes and the analysis were completed by December 2022. The demographic data were obtained through the Registered Persons Database (RPDB). Unique encoded identifiers were used to link multiple datasets together.

This analysis included all residents 65 years of age and older. The age variable was grouped as 65–69, 70–74, 75–79, 80–84, and ≥ 85 years. The two outcome variables included presence of pressure ulcers and dehydration at each assessment (see S1 Table for definitions). Number of assessments for residents varied from 1 to 8 during the study period, and residents without any assessment were excluded. In addition to sex and age of residents, the following variables were included in the analysis: region or location of the LTC home as the Central, Southwest, East, or North of Ontario; ownership category of the LTC home as municipal, non-profit, or private (for-profit); and Changes in Health, End-stage disease Symptoms and Signs (CHESS) comorbidity score. The CHESS score varies from 0 (no instability in health) to 5 (high unstable health).

The project was approved by the ICES Privacy and Legal Office. ICES is a prescribed entity under section 45 of Ontario’s *Personal Health Information Protection Act* (2004). Section 45 authorizes ICES to collect personal health information without consent for the purpose of analysis or compiling statistical information with respect to the management of, evaluation, or monitoring of the allocation of resources or planning for all or part of the health system. Projects conducted under section 45 do not require informed consent. The Hamilton Integrated Research Ethics Board (HiREB) reviewed and approved the study (HiREB#11526).

### Statistical analysis

We used descriptive statistics to summarize the sample characteristics. The reported descriptive statistics include mean and standard deviation (SD) for the CHESS comorbidity score as the only continuous variable and frequency and percentage for the other variables. The CHESS score for each resident was calculated as the mean of CHESS over the number of assessments. We used an independent two-sample t-test to compare CHESS comorbidity scores between those with and without pressure ulcers and those with and without dehydration. We used a Chi-squared test to compare different levels of each categorical variable for each outcome variable.

To incorporate multiple assessments for each resident, we used a mixed-effect multilevel logistic regression analysis to identify factors associated with pressure ulcers and dehydration. In this hierarchical model, multiple assessments are nested within residents and residents are nested within LTC homes. As a result, variables representing resident and LTC home were used as the random components of the model to accommodate the intraclass correlation within resident and LTC home. The variables in the multilevel logistic regression included sex, age group, CHESS score, region, and type of LTC home ownership. In addition, we noted a quadratic association between CHESS scores and risk of pressure ulcers and dehydration, which indicated introduction of squared values of CHESS score as a variable in our models. We used a significance level of *α* = 0.05 in all analysis. Only less than 1% of some variables were missing, and a case-wise deletion approach was used in analysis. All statistical analyses were conducted using Stata/MP 15.1 (Stata Corporation, College Station, TX).

## Results

Analysis included 625 LTC homes in Ontario with 96,590 residents, of which 30,512 (31.6%) were male and 66,078 (68.4%) were female. The majority of residents (54,585; 56.5%) were 85 years of age or older and 50,903 (52.7%) were living in private-owned LTC homes (Table 1). More residents showed symptoms of pressure ulcers than symptoms of dehydration. The distribution of these conditions based on demographic variables showed men were more prone to pressure ulcers and women had more symptoms of dehydration.

**Table 1:** Distribution of pressure ulcer and dehydration based on demographics from January 2019 to February 2020.

Category	Total	Pressure Ulcer		Dehydration	
		n (%)	p-value	n (%)	p-value
**Sex**					
Male	30,512	6150 (20.2)	<0.001	4767 (15.6)	<0.001
Female	66,078	12,136 (18.4)		11,894 (18.0)	
**Age group**					
65–69 years	4982	831 (16.7)	<0.001	649 (13.0)	<0.001
70–74 years	7789	1357 (17.4)		1088 (14.0)	
75–79 years	11,480	1925 (16.8)		1718 (15.0)	
80–84 years	17,754	3301 (18.6)		2799 (15.8)	
≥85 years	54,585	10,872 (19.9)		10,407 (19.1)	
**Age**: Mean (SD)	84.8 (8.2)	-	<0.001	-	<0.001
Without condition^b^	-	84.7 (8.2)		84.6 (8.2)	
With condition^b^	-	85.5 (8.2)		86.0 (8.0)	
**CHESS**^a^: Mean (SD)	0.94 (0.69)	-	<0.001	-	<0.001
Without condition^b^	-	0.88 (0.66)		0.86 (0.65)	
With condition^b^	-	1.16 (0.76)		1.31 (0.74)	
**Region**					
Central	25,376	4026 (15.9)	<0.001	2471 (9.7)	<0.001
Southwest	32,741	7038 (21.5)		7730 (23.6)	
East	26,225	4689 (17.9)		3391 (12.9)	
North	12,173	2516 (20.7)		3061 (25.1)	
**Long-term care home ownership**					
Municipal	19,894	4246 (21.3)	<0.001	3974 (20.0)	<0.001
Non-profit	25,661	4973 (19.4)		4723 (18.4)	
Private	50,903	9060 (17.8)		7851 (15.4)	
**Total**	**96,590**	**18,286 (18.9)**	-	**16,661 (17.3)**	-

^a^ Changes in Health, End-stage disease and Symptoms and Signs scale.

^b^ The condition indicates pressure ulcer or dehydration.

In univariate analysis increased age was correlated with an increased risk of pressure ulcers and dehydration (p<0.001). The lowest rates of both conditions were in the Central region of Ontario and the highest rates were in the Southwestern region. The differences in rates were highly significant between the regions (p<0.001). Analysis also showed significant differences between rates of pressure ulcers and dehydration based on LTC home ownership. Incidence rates for both conditions were higher in municipal owned homes compared to private owned homes.

### Factors associated with pressure ulcers

Multilevel mixed-effect logistic models were used to show factors associated with pressure ulcers and dehydration. The effect size for each level of a factor is shown with odds ratio (OR) and 95% confidence interval. All the demographic variables were significantly associated with pressure ulcers (Table 2). Female residents were 34% less likely to be diagnosed with pressure ulcers than male residents (OR = 0.66 [95% CI: 0.62, 0.70]). There was no significant differences between pressure ulcers and age for groups younger than 85 years. However, the likelihood of pressure ulcers for residents 85 years and older increased 15% compared to the youngest age group (OR = 1.15 [95% CI: 1.01, 1.32]). The results also showed a quadratic association between pressure ulcers and CHESS score.

**Table 2:** Association between demographics and pressure ulcers.

Variable	With Pressure Ulcer n (%)	Without Pressure Ulcer n (%)	OR^a^ (95% CI)	P-value
**Sex**				
Male	6150 (20.2)	24,362 (79.8)	Reference group	-
Female	12,136 (18.4)	53,942 (81.6)4982	0.66 (0.62, 0.70)	<0.001
**Age group**				
65–69 years	831 (16.7)	4151 (83.3)	Reference group	-
70–74 years	1357 (17.4)	6432 (82.6)	1.08 (0.92, 1.28)	0.361
75–79 years	1925 (16.8)	9555 (83.2)	0.92 (0.79, 1.08)	0.308
80–84 years	3301 (18.6)	14,453 (81.4)	1.07 (0.92, 1.24)	0.370
≥85 years	10,872 (19.9)	43,713 (80.1)	1.15 (1.01, 1.32)	0.043
**CHESS**^b^ **scale**				
CHESS: Mean (SD)	0.88 (0.66)	1.16 (0.76)	1.76 (1.69, 1.84)	<0.001
CHESS-Sq^c^	-	-	1.02 (1.01, 1.03)	<0.001
**Region**				
Central	4026 (15.9)	21,350 (84.1)	Reference group	-
Southwest	7038 (21.5)	25,703 (78.5)	1.34 (1.13, 1.60)	0.001
East	4689 (17.9)	21,536 (82.1)	1.06 (0.89, 1.25)	0.540
North	2516 (20.7)	9657 (79.3)	1.13 (0.90, 1.41)	0.286
**Long-term care home ownership**				
Private	9060 (17.8)	41,843 (82.2)	Reference group	-
Municipal	4246 (21.3)	15,648 (78.7)	1.54 (1.26, 1.89)	<0.001
Non-profit	4973 (19.4)	20,688 (80.6)	1.21 (1.01, 1.45)	0.040

^a^ Odds ratio.

^b^ Changes in Health, End-stage disease and Symptoms and Signs.

^c^ CHESS scale squared.

Among the regions, the highest rate of pressure ulcers was in Southwestern Ontario. The likelihood of LTC residents developing this condition was 34% higher in this region than in Central Ontario. There was no significant difference between the Eastern, Northern, and Central regions. Yet there were significant differences based on home ownership. Compared to homes with private (for-profit) ownership, the likelihood of pressure ulcers was 54% higher for residents in municipal homes (OR = 1.54 [95% CI: 1.26, 1.89]) and 21% higher for residents in non-profit homes (OR = 1.21 [95% CI: 1.01, 1.45]).

### Factors associated with dehydration

Factors associated with dehydration are shown in Table 3. There was a significant association between sex and dehydration where the likelihood of dehydration was 13% higher for female residents [OR = 1.13 (95%CI: 1.06, 1.20)]. Similar to pressure ulcers, residents 85 years and older were more likely to suffer from dehydration compared to the youngest age groups [OR = 1.34 (95%CI: 1.16, 1.53)]. In addition, both linear and quadratic components of CHESS score were highly associated with dehydration (p <0.001). The highest risk of dehydration was observed in Southwestern Ontario [OR = 1.42 (95%CI: 1.09, 1.86)]. In addition, risk of dehydration in municipal homes were more than twice compared to private homes (OR = 2.06 [95% CI: 1.18, 3.59]).

**Table 3:** Association between demographics and dehydration.

Variable	With Dehydration n (%)	Without Dehydration n (%)	OR^a^ (95% CI)	P-value
**Sex**				
Male	4767 (15.6)	25,745 (84.4)	Reference group	-
Female	11,894 (18.0)	54,184 (82.0)	1.13 (1.06, 1.20)	<0.001
**Age group**				
65–69 years	649 (13.0)	4333 (87.0)	Reference group	-
70–74 years	1088 (14.0)	6701 (86.0)	1.04 (0.88, 1.23)	0.676
75–79 years	1718 (15.0)	9762 (85.0)	1.05 (0.90, 1.23)	0.504
80–84 years	2799 (15.8)	14,955 (84.2)	1.08 (0.93, 1.25)	0.315
≥85 years	10,407 (19.1)	44,178 (80.9)	1.34 (1.16, 1.53)	<0.001
**CHESS**^b^ **scale**				
CHESS: Mean (SD)	84.6 (8.2)	86.0 (8.0)	1.87 (1.78, 1.96)	<0.001
CHESS-Sq^c^	-	-	1.14 (1.13, 1.15)	<0.001
**Region**				
Central	2471 (9.7)	22,905 (90.3)	Reference group	-
Southwest	7730 (23.6)	25,011 (76.4)	1.42 (1.09, 186)	0.009
East	3391 (12.9)	22,834 (87.1)	1.03 (0.81, 1.31)	0.802
North	3061 (25.1)	9112 (74.9)	1.38 (0.98, 1.94)	0.067
**LTC home ownership**				
Private	7851 (15.4)	43,052 (84.6)	Reference group	-
Municipal	3974 (20.0)	15,920 (80.0)	2.06 (1.18, 3.59)	0.011
Non-profit	4723 (18.4)	20,938 (81.6)	1.15 (0.71, 1.85)	0.564

^a^ Odds ratio.

^b^ Changes in Health, End-stage disease and Symptoms and Signs.

^c^ CHESS scale squared.

## Discussion

This retrospective study used administrative organizational-level and resident-level data to examine factors associated with pressure ulcers and dehydration among LTC residents in Ontario, Canada. Results indicated that close to one-fifth of residents were dehydrated (17.3%) or had a pressure ulcer (18.9%) during the study period. Namasivayam-MacDonald et al. [19] likewise found that daily fluid intake was considerably less than the recommended amount for residents of LTC homes in four provinces, including Ontario. However, in contrast to our findings, Woo et al. [29] reported a one-year pressure ulcer prevalence rate of 8.9% in LTC homes in Ontario. The presence of pressure ulcers and dehydration vary according to age, sex, geographical region, and home ownership. Our study corroborates existing evidence that advanced age is significantly associated with the presence of pressure ulcers and dehydration for both men and women [4, 19]. However, we found men were more likely to present with a pressure ulcer while women were more likely to exhibit symptoms of dehydration.

The study findings also demonstrate significant differences between pressure ulcers and dehydration based on LTC home ownership with the presence of both conditions being higher in municipal and not-for-profit homes compared to for-profit homes. These findings contrast with other literature that demonstrates poor outcomes in for-profit homes compared to not-for-profit and government-run homes [8, 9, 27, 30–32]. Systematic reviews have reported inconsistent findings related to the relationship between home ownership and quality indicators such as pressure ulcers, dehydration, falls and infection rates [33, 34]. In a study conducted in the Republic of Korea, researchers used pressure ulcers as a proxy for quality of care to understand the impact of increased privatization in the LTC sector. Results revealed that residents in private LTC homes had an appreciably higher risk of pressure ulcers than those in government-run facilities [35]. In contrast, a Canadian study found higher rates of pressure ulcers and dehydration in government-run homes compared to for-profit homes [8]. However, the researchers indicated that the odds of dying from one of these conditions was greater among residents in for-profit homes [8].

Consistent findings in the literature relate to the relationship between staffing levels and home ownership. Studies show that publicly owned facilities have higher staffing levels and better health outcomes than privately owned facilities [27, 28]. For example, research from the US has demonstrated that increased registered nurse (RN) staff hours result in decreased pressure ulcers, catheterization, urinary tract infections, ADL decline, nutritional supplements, and hospitalizations [36, 37]. Similarly, an increase in licensed practical nurse (LPN) staff hours is associated with decreases in quality care indicators including pressure ulcers and use of physical restraints [38]. In Canada, residents living in government-run facilities receive more direct care per day compared to those living in for-profit homes [30, 31]. Given the lack of consistency in staffing levels across different types of homes there may be underreporting of these quality indicators in homes with lower capacity [31]. Further investigation is required to assess the relationship between home ownership, staffing levels and quality of care.

### Limitations

There are several limitations to the data set used in our analysis. As with all administrative databases, this study is subject to coding error. The nature of the study means we cannot make predictions or infer causality; we can only report associations. Although RAI reporting is mandatory, the reporting is not consistent/standardized over time.

## Conclusions

Pressure ulcers and dehydration are preventable conditions that warrant attention from policymakers to ensure quality of care and resident safety are prioritized. Results of this study indicate higher rates of pressure ulcers and dehydration in municipal and not-for-profit homes compared to for-profit homes. However, the significant differences observed require further investigation to identify the most relevant factors in preventing these two conditions.

## Supporting information

S1 Table Definitions of pressure ulcer and dehydration. (DOCX)

S1 File Statistical codes. (TXT)
