# Supplementary material for: Factors associated with pressure ulcer and dehydration in long-term care settings in Ontario, Canada
Source: PLoS One. 2024 Jan 31;19(1):e0297588. doi: 10.1371/journal.pone.0297588 (PMC10830047; doi:10.1371/journal.pone.0297588)
Supplement: S1 Table — (DOCX) [file pone.0297588.s001.docx]

**S1 Table.** Definitions of pressure ulcer and dehydration

| **Variable** | **RAI-MDS definition** |
| --- | --- |
| Pressure ulcer | Pressure ulcers are defined as any lesions caused by pressure resulting in damage of underlying tissue. They present across four stages:  Stage 1: A persistent area of skin redness (without a break in the skin) that does not disappear when pressure is relieved.  Stage 2: A partial thickness loss of skin layer that presents clinically as an abrasion, blister or shallow crater.  Stage 3: A full thickness of skin is lost, exposing the subcutaneous tissues. It presents as a deep crater with or without undermining adjacent tissue.  Stage 4: A full thickness of skin and subcutaneous tissue is lost, exposing muscle or bone.  The occurrence of pressure ulcers at any stage that presented within the last seven days was recorded by the health professional. |
| Dehydration | Dehydration is defined as the point when output exceeds intake. Health professionals are advised to check this item if the resident has two or more of the following indicators:   - Resident usually takes in less than the recommended 2,500 mL of fluids daily (water or liquids in beverages and water in food). - Resident has clinical signs of dehydration. - Resident’s fluid loss exceeds the amount of fluids they take in (e.g., loss from vomiting, fever, diarrhea that exceeds fluid replacement). |

^a^. Morris, J. N., Hawes, C., Mor, V., Phillips, C., Fries, B. E., Nonemaker, S. & Murphy, K. (2012). Resident Assessment Instrument (RAI) RAI-MDS 2.0 User's Manual, Canadian Version, Washington, DC: interRAI 2010.
